# Methylene blue and normobaric hyperoxia combination therapy in experimental ischemic stroke

**DOI:** 10.1002/brb3.478

**Published:** 2016-05-04

**Authors:** Pavel Rodriguez, Jiang Zhao, Brian Milman, Yash Vardhan Tiwari, Timothy Q. Duong

**Affiliations:** ^1^ Research Imaging InstituteUniversity of Texas Health Science CenterSan AntonioTexas; ^2^ Department of RadiologyUniversity of Texas Health Science CenterSan AntonioTexas; ^3^ Department of Anatomy and EmbryologyPeking University Health Science CenterBeijingChina; ^4^ Department of Biomedical EngineeringUniversity of TexasSan AntonioTexas

**Keywords:** Cerebral ischemia, combination therapy, methylene blue, normobaric hyperoxia, stroke

## Abstract

**Introduction:**

Ischemic stroke is a global burden that contributes to the disability and mortality of millions of patients. This study aimed to evaluate the efficacy of combined MB (methylene blue) and NBO (normobaric hyperoxia) therapy in experimental ischemic stroke.

**Methods:**

Rats with transient (60 min) MCAO (middle cerebral artery occlusion) were treated with: (1) air + vehicle (*N* = 8), (2) air + MB (*N* = 8), (3) NBO + vehicle (*N* = 7), and (4) NBO + MB (*N* = 9). MB (1 mg/kg) was administered at 30 min, again on days 2, 7, and 14 after stroke. NBO was given during MRI (30–150 min) on day 0, and again 1 h each during MRI on subsequent days. Serial diffusion, perfusion and T2 MRI were performed to evaluate lesion volumes. Foot‐fault and cylinder tests were performed to evaluate sensorimotor function.

**Results:**

The major findings were: (1) NBO + MB therapy showed a greater decrease in infarct volume compared to NBO alone, but similar infarct volume compared to MB alone, (2) NBO + MB therapy accelerated sensorimotor functional recovery compared to NBO or MB alone, (3) Infarct volumes on day 2 did not change significantly from those on day 28 for all four groups, but behavioral function continued to show improved recovery in the NBO + MB group.

**Conclusions:**

These findings support the hypothesis that combined NBO + MB further improves functional outcome and reduces infarct volume compared to either treatment alone and these improvements extended up to 28 days.

## Introduction

MB (Methylene blue) is a United States Food and Drug Administration grandfathered pharmaceutical that is safely used to treat methemoglobinemia and cyanide poisoning (Clifton and Leikin 2003; Scheindlin 2008). Low‐dose MB (1–5 mg/kg) has been shown to cycle electrons to cytochrome C oxidase in the mitochondrial electron transport chain, which leads to increased downstream production of adenosine triphosphate (ATP) and decreased free radical production (Clifton and Leikin 2003; Zhang et al. 2006). In recent years, a few studies have found MB to be neuroprotective in treating acquired brain injuries and neurodegenerative diseases. Low‐dose MB has been reported to reduce neurobehavioral impairment in animal models of optic neuropathy (Rojas et al. 2009a), Parkinson's disease (Ishiwata et al. 2006; Rojas et al. 2009b), Alzheimer's disease (Callaway et al. 2002; Riha et al. 2011; Auchter et al. 2014), and traumatic brain injury (Watts et al. 2014). Low‐dose MB also prolongs the perfusion–diffusion mismatch (i.e., ischemic penumbra) (Rodriguez et al. 2014) and decreases infarct volume and behavioral deficit (Shen et al. 2013). MB‐mediated neuroprotection has been linked to enhanced autophagy (Jiang et al. 2015), inhibited apoptosis in the ischemic tissue (Jiang et al. 2015), and augmented mitophagy (Di et al. 2015). These positive neuroprotective effects are consistent with MB's energy‐enhancing and antioxidant properties.

NBO (Normobaric hyperoxia) treatment in ischemic stroke improves tissue oxygenation (Shin et al. 2007), aerobic metabolism (Singhal et al. 2007), and reduces blood‐brain barrier disruption (Liu et al. 2009), free radical damage (Yuan et al. 2010), and peri‐infarct depolarization (Shin et al. 2007). A few NBO clinical trials have demonstrated transient improvement in diffusion weighted imaging (DWI) abnormalities and improved NIHSS scores in humans (Kidwell et al. 2000; Singhal et al. 2004; Wu et al. 2012). However, some animal studies found NBO treatment to yield worse outcomes, such as increased white matter necrosis and infarct volumes (Balentine 1968; Mickel et al. 1987, 1990). A recent phase II clinical trial (NCT00414726) in which NBO was given within the first 24 h was terminated early due to a significant increase in the number of death in the intervention NBO group compared to control (Cornet et al. 2013). Another clinical trial also showed that NBO increased mortality in patients with moderate to severe strokes when given in the first 24 h (Ronning and Guldvog 1999). Despite questionable NBO efficacy in human stroke, NBO treatment was among the most effective therapy in reducing infarct volume in animal models. As such, a number of combination therapy with other neuroprotective agents (such as antioxidant or free radical scavengers) have also been explored (Albers 1999; Jin et al. 2013; Parmar et al. 2015).

Perfusion and diffusion MRI are widely used to distinguish reversible from irreversible ischemic brain injury, and to guide acute stroke treatment in preclinical and clinical settings (Schlaug et al. 1999). When CBF (cerebral blood flow) drops below a critical threshold, energetic failure results and the ADC (apparent diffusion coefficient) of water in the tissue starts to decrease (Moseley et al. 1990). Diffusion‐weighted MRI, in which image contrast is based on water ADC, can detect ischemic injury within minutes after onset, whereas computed tomography and other imaging modalities fail to detect stroke injury for at least a few hours (Moseley et al. 1990). T2 MRI is also used to delineate infarct tissue and vasogenic edema (Shen et al. 2003, 2004, 2005). With the improved acquisition speed and sensitivity of MRI technologies, it becomes possible to identify patients likely to benefit from acute stroke treatment (i.e., a perfusion–diffusion mismatch is present), monitor treatment efficacy, optimize treatment time window, amongs others (Albers 1999). There is renewed interest in exploring novel treatment strategies, including combination therapies, in ischemic stroke.

Given that MB alone and NBO alone have been shown to have therapeutic effects in animal models of ischemic stroke, we reasoned that the combination treatment of MB + NBO has a high likelihood of reducing oxygen free radical production while sustaining energy production and tissue oxygenation, thereby expanding the treatment time window and improving outcomes. The combination therapy could prolong the viability of the tissue at risk for infarction via complementary mechanisms. Specifically, MB could increase the time window of the tissue at risk by its effect on mitochondrial function and the potential rescue of free radical injury, and NBO would aid the same tissue by its effects on oxygenation. Additionally, other pathways targeted by MB, which ultimately inhibit apoptosis and have other pleiotropic effects, could also aid in the chronic behavioral recovery of animals, and rescue the negative effects of prolonged NBO administration.

The goal of this study was thus to evaluate the efficacy of MB, NBO, and combined MB + NBO treatment in an established rat MCAO stroke model using noninvasive MRI and behavioral assessment. We tested the hypothesis that combined MB + NBO treatment further reduces infarct volume and functional deficits compared to air, MB or NBO treatment alone. Multimodal MRI included perfusion, diffusion and T2 MRI for animal selection and to longitudinally measure the lesion evolution in the same animal. Behavioral tests included foot‐fault and forelimb asymmetry to assess sensorimotor function.

## Methods

All experimental procedures were approved by the Institutional Animal Care and Use Committees of the University of Texas Health Science Center San Antonio. Male Sprague‐Dawley rats (250–305 g, *n* = 60) were purchased from Charles River Laboratories (Wilmington, MA). Animals were allowed free access to food and water before and after surgery, and were maintained under diurnal lighting conditions. Four groups of animals were studied: (1) air + vehicle (*N* = 11), (2) air + MB (*N* = 11), (3) NBO + vehicle (*N* = 11), and (4) NBO + MB (*N* = 14).

### Inclusion and exclusion criteria

One animal was excluded due to hemorrhagic transformation and two animals died prior to 28 days endpoints. Animals were excluded a priori if the initial lesion volume at 30 min was less than 115 mm^3^ or greater than 290 mm^3^, which ensured involvement of cortex and subcortex in all animals and avoided large pathological diffusion abnormality. There were 3, 2, 3, and 2 animals excluded, respectively, from groups 1–4, respectively, due to infarct volume outside of inclusion criteria. As a result, the sample sizes in the final analysis included: (1) air + vehicle (*N* = 8), (2) air + MB (*N* = 8), (3) NBO + vehicle (*N* = 7), and (4) NBO + MB (*N* = 9).

### Animal procedures

Transient (60 min) right hemisphere ischemia was induced using 0.35–0.37 intraluminal silicon rubber‐coated filaments (Doccol Corporation, Sharon, MA) to occlude the right middle cerebral artery. Animals were mechanically ventilated and maintained at 1.2–1.5% isoflurane in room air while in the MRI scanner. A stereotactic headset was used to place the animal in the MRI scanner. End‐tidal CO_2_ was maintained using a SurgiVet V9004 Capnographer (Smiths Medical, Norwell, MA). Heart rate, arterial oxygen saturation, and breathing rate were monitored using a MouseOx system (Star Life Sciences, Oakmont, PA). The core temperature was maintained at 37 ± 0.5°C using a rectal probe sensor connected to water bath‐water blanket feedback loop. Physiological measurements were recorded and maintained within normal ranges throughout the experiment.

**Figure 1:**
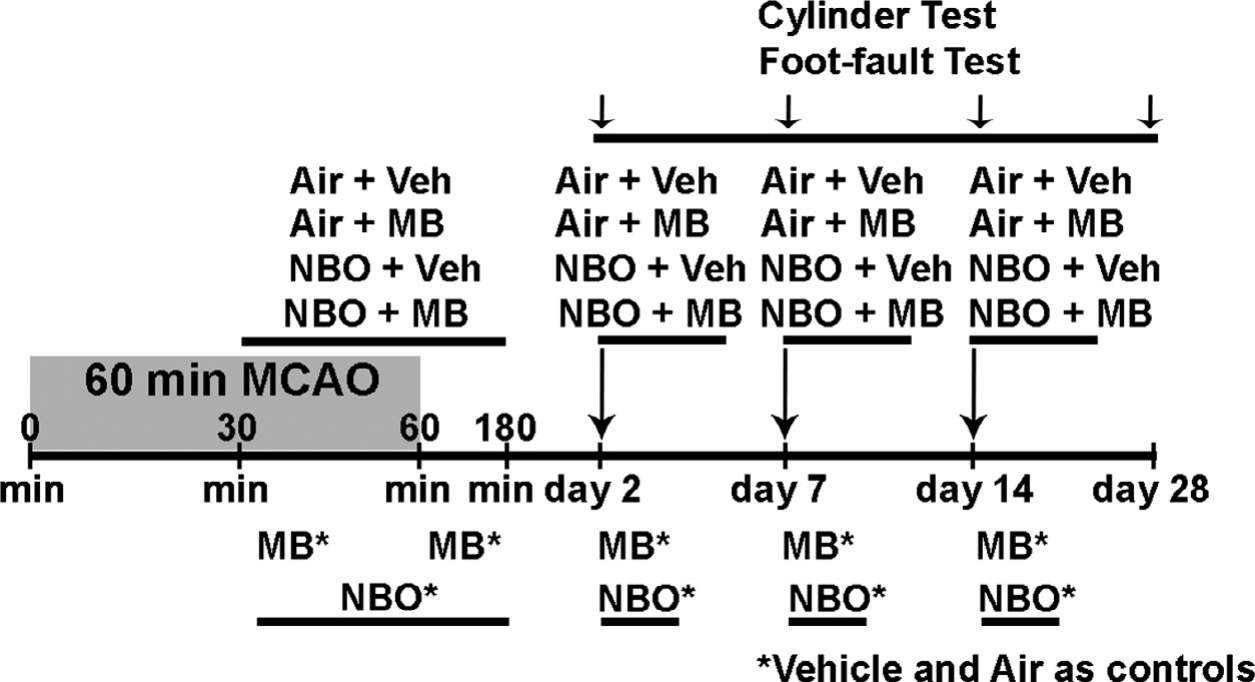
Sixty minutes MCAO (middle cerebral artery occlusion). Schematic representation of the four 60 min MCAO experimental groups. The infusion of MB (methylene blue) (1 mg/kg) or Vehicle was given over 25–30 min beginning at 30 min and immediately after reperfusion at 60 min. NBO or Air was administered for 150 min after the baseline scans before and after reperfusion. MB (1 mg/kg) or Vehicle with 45–60 min NBO or Air was administered on Day 2, 7, and 14. Functional assessment was also performed on day 2, 7, 14, and 28.

A randomization scheme was used to allocate the type of intervention prior to stroke surgery, and the surgeon and data analysts were blinded to the type of intervention. The same person performed all surgeries. Either vehicle (heparinized normal saline) or 1 mg/kg USP MB (10 mg/kg American Regent, Shirley, NY) was infused via tail vein over 30 min using an MRI compatible pump (Harvard Apparatus, Holliston, MA). Concurrent room air or normobaric oxygen (2.5 L/min) was administered after the baseline scans over the following total 150 min. An ADC scan was acquired 57–60 min (i.e., immediately before reperfusion), and the animal was removed from the MRI scanner and stereotactic headset for removal over the filament from the right MCA. NBO (2.5 L/min) or air administration was not interrupted by the procedure. After reperfusion, 1 mg/kg MB or vehicle was administered over 30 min via tail vein perfusion. The animals were allowed to recover from anesthesia and placed back in their cage. On days 2, 7, and 14, 1 mg/kg MB or vehicle was administered via intraperitoneal injection after the behavioral tests, but prior to follow‐up imaging in combination with NBO (2.5 L/min) or room air during the length of the follow‐up scans (approximately 45–60 min including setup time). Figure 1 demonstrates a schematic representation of the experimental procedures.

### Functional assessments

Behavioral assessments were conducted on days 2, 7, 14, and 28 using the validated foot‐fault and cylinder tests as previously described (Hernandez and Schallert 1988; Watts et al. 2014, 2015). The foot‐fault test is a measure of motor impairment. The test consists of free movement in a grid‐like environment. A foot‐fault is counted when there is incorrect placement of an impaired forward limb which leads to a slip (called foot‐fault). The cylinder test measures somatosensory and motor capacity. The animal is placed in a Plexiglas cylinder and allowed to explore the vertical surfaces with the forelimbs and rearing. Impairment of the right hemisphere MCA distribution results in contralateral decreased use of the affected forelimb. The day 2 cylinder test values could not be obtained for the cylinder test due to animal inactivity. Animals that attempted less than 25 steps in the foot‐fault test, or 30 forepaw touches in the cylinder test, were excluded from the analysis due to lack of test reliability. Baseline surgeries, follow‐up imaging and behavioral assessments were conducted during normal daytime working hours.

### MRI experiments

MRI experiments were conducted using a Bruker Biospec 7 Tesla/30 cm scanner with a 76 G/cm BGA12S gradient insert (Billerica, MA) using custom made brain imaging and single loop perfusion neck coils (Shen et al. 2003, 2004, 2005). ADC and CBF maps were acquired at 30 and 60 minutes, and days 2, 7, 14, and 28. T2‐weighted maps were acquired on day‐2, 7, 14 and 28.

Apparent diffusion coefficient was measured using spin‐echo diffusion‐weighted echo‐planar imaging and two b values of 4 and 1200 sec/mm^2^ (32) where diffusion gradients were applied separately along the x, y, and z directions, separation between diffusion gradient Δ = 9 msec, diffusion gradient duration *δ *= 3 msec, seven 1.5 mm thick slices, TR (repetition time) = 3 sec, TE (echo time) = 30 msec, matrix = 96 × 96 (reconstructed to 128 × 128), FOV (field of view) = 25.6 × 25.6 mm, 90° flip angle, and 2 transients for signal averaging.

Cerebral blood flow was measured using continuous arterial spin labeling with gradient‐echo‐planar imaging, a 2.7‐sec square radiofrequency pulse applied to the labeling coil followed by a 250 msec post‐labeling delay and TE = 10.2 msec. Seven 1.5 mm thick slices, TR = 3 sec, matrix = 96 × 96 (reconstructed to 128 × 128), FOV = 25.6 × 25.6 mm and 90° flip angle.

T_2_: T_2_‐weighted images were acquired using a fast spin‐echo pulse sequence with four effective TEs 25, 40, 75 and 120 msec), TR = 3 sec (90^o^ flip angle), matrix = 128 × 128, FOV = 25.6 × 25.6 mm, seven 1.5 mm thick slices, echo train length = 4, and 4 signal averages.

### Data analysis

Lesion volumes were measured using Stimulate 8.0.1 (University of Minnesota). T2 maps were calculated for days 2, 7, 14, and 28. ADC values were calculated using upper limit signal thresholds for the abnormal hemisphere that were calculated from an region of interest (ROI) drawn in the normal hemisphere minus three standard deviation of the noninfarcted tissue as previously described (Rodriguez et al. 2014). Therefore, only regions that differed by more than 99.7% signal value were included in the infarct volume. CBF volumes were defined using the mean normal hemisphere CBF signal minus one times the standard deviation of the noninfarcted hemisphere in order to detect subtle perfusion volume changes. The mean T2 value plus two times the standard deviation of the normal hemisphere was used as the threshold for T2 volumes. Edema‐correction was only applied for day 2 measurements and was calculated as infarct volume – (right hemisphere – left hemisphere) (Meng et al. 2004).

### Statistical analysis

A one‐way ANOVA with Tukey's post hoc analysis was used for comparison across different groups at 30 and 60 min using the ADC and CBF acquisitions. A factorial repeated measures ANOVA with post hoc analysis and multivariate testing (Wilk's Lambda) was used to compare the within and between group interactions over time for days 2, 7, 14, and 28 T2‐defined infarct volumes. Standard assumptions for homogeneity of variance (Levene's test) and equality of covariance matrices (Box's Test) were met. Lesion volumes are expressed as means ± standard error of the mean. Statistical analysis was conducted in SPSS Statistics v. 22 (International Business Machines Corporation, Armonk, NY). A *P* value of 0.05 was used for statistical significance.

## Results

### Physiological parameters

For all four groups prior to initiating any intervention, the baseline heart rate (390–430 bpm), arterial oxygen saturation (92–95%), end‐tidal CO_2_ (3.2–3.4%) and temperature (36.9–37.3°C) were within normal physiological ranges and were not statistically different from each other. Arterial oxygen saturation increased in the hyperoxia groups NBO + Vehicle and NBO + MB as expected. The remaining physiologic measurements did not differ between groups after intervention.

### Longitudinal neuroimaging assessment

**Figure 2:**
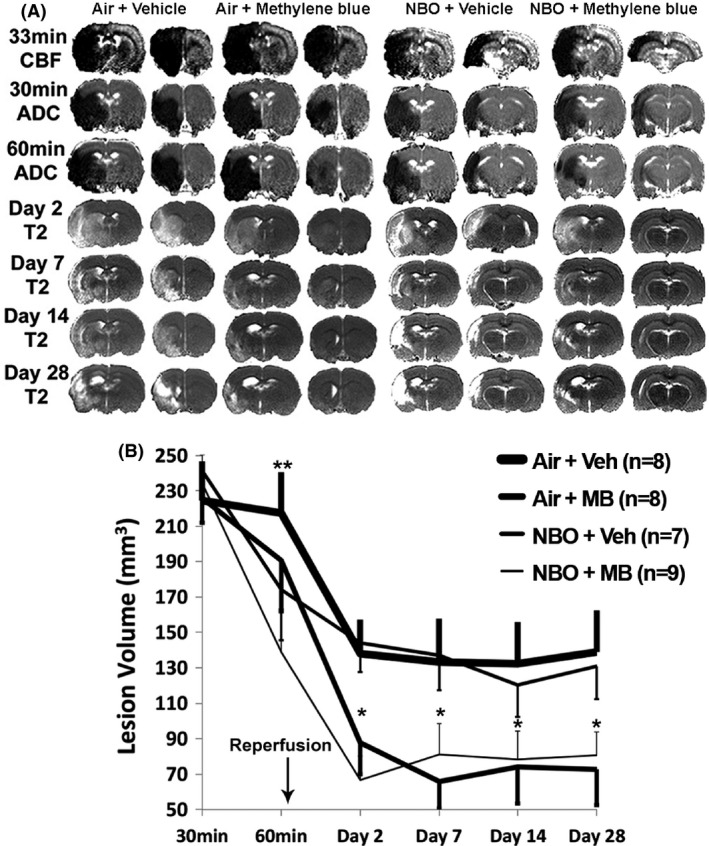
Longitudinal neuroimaging assessment. (A) Representative cerebral blood flow and ADC (apparent diffusion coefficient) maps on day 0, and T2 volumes for the four follow‐up time points for the Air + Vehicle, Air + MB, NBO + Vehicle, and NBO + MB. There is gradual resolution of edema and increase in encephalomalacia over time from day 0 (B) Evolution of ADC measurements over 30 min for the four experimental groups, and T2 measurements on day 2, 7, 14, and 28. At 60 min, there were significant within‐group decreases in infarct volumes for the NBO + Vehicle (**P = 0.03) and NBO + MB (**P < 0.002) relative to 30 min volumes, but not amongs groups (*P* = 0.15). *The decrease in infarct volumes was significant across time in the NBO + Vehicle versus Air + MB (*P* = 0.039), and NBO + Vehicle versus NBO + MB (*P* = 0.03), but not between the NBO + Vehicle versus Air + Vehicle (*P* = 0.9).

Representative CBF maps at 33 min, ADC maps at 30 and 60 min, and T2 maps for days 2, 7, 14, and 28 are shown in Figure 2A and the group lesion volume data are shown in Figure 2B. Abnormal CBF was detected at 30 min after MCAO, and were not statistically different amongs all four groups at 30 min. There was also no significant difference in within‐group CBF associated with the 25–30 min intervention (*P* > 0.05, data not shown).

At 30 min, baseline ADC lesion volumes for the Air + Vehicle, Air + MB, NBO + Vehicle, and NBO + MB groups (224 ± 22, 226 ± 17, 242 ± 12, and 234 ± 16 mm^3^, respectively) were not significantly different amongs all groups (*P* = 0.9). At 30 min, the corresponding baseline CBF deficit volumes were 346 ± 9, 341 ± 22, 328 ± 121, and 337 ± 13 mm^3^, respectively (*P* = 0.9). The CBF deficit volumes were significantly different from ADC lesion volumes for each group (*P* < 0.05), indicative of the presence of perfusion–diffusion mismatch.

At 60 min (before reperfusion and after initiating intervention), all three treatment groups showed reduced ADC lesion volumes compared to Air + Vehicle, and the NBO + Vehicle group had larger ADC lesion volume reduction than Air + MB, and the NBO + MB group showed the largest decrease in ADC defined lesion volume. This is depicted by the normalization in ADC signal in the right hemisphere. Although the abnormal ADC volumes at 60 min were not significantly different across groups (*P* = 0.145, one‐way ANOVA), within‐group paired comparison with baseline ADC volumes was significant for the Air + MB, NBO + Vehicle, and NBO + MB groups (191 ± 28, 174 ± 63, and 139 ± 22 mm^3^, respectively, *P* = 0.002 to 0.04).

On day 2, the infarct volumes for the Air + Vehicle, Air + MB, NBO + Vehicle, and NBO + MB groups were 138 ± 19, 88 ± 18, 144 ± 16, and 67 ± 13 mm^3^, respectively. On days 2–28, the NBO + Vehicle group leveled off at a comparatively larger T2 lesion volume that was not significantly different from the Air + Vehicle group. By contrast, Air + MB and NBO + MB leveled off to a significantly smaller T2 lesion volume on day 2 to 28 compared to the other groups across time using repeated measures ANOVA (*P* = 0.03 to 0.04). On day 28, the infarct volumes for the Air + Vehicle, Air + MB, NBO + Vehicle, and NBO + MB groups were 139 ± 23, 73 ± 20, 131 ± 19, and 81 ± 14 mm^3^, respectively.

### Longitudinal functional assessment

**Figure 3:**
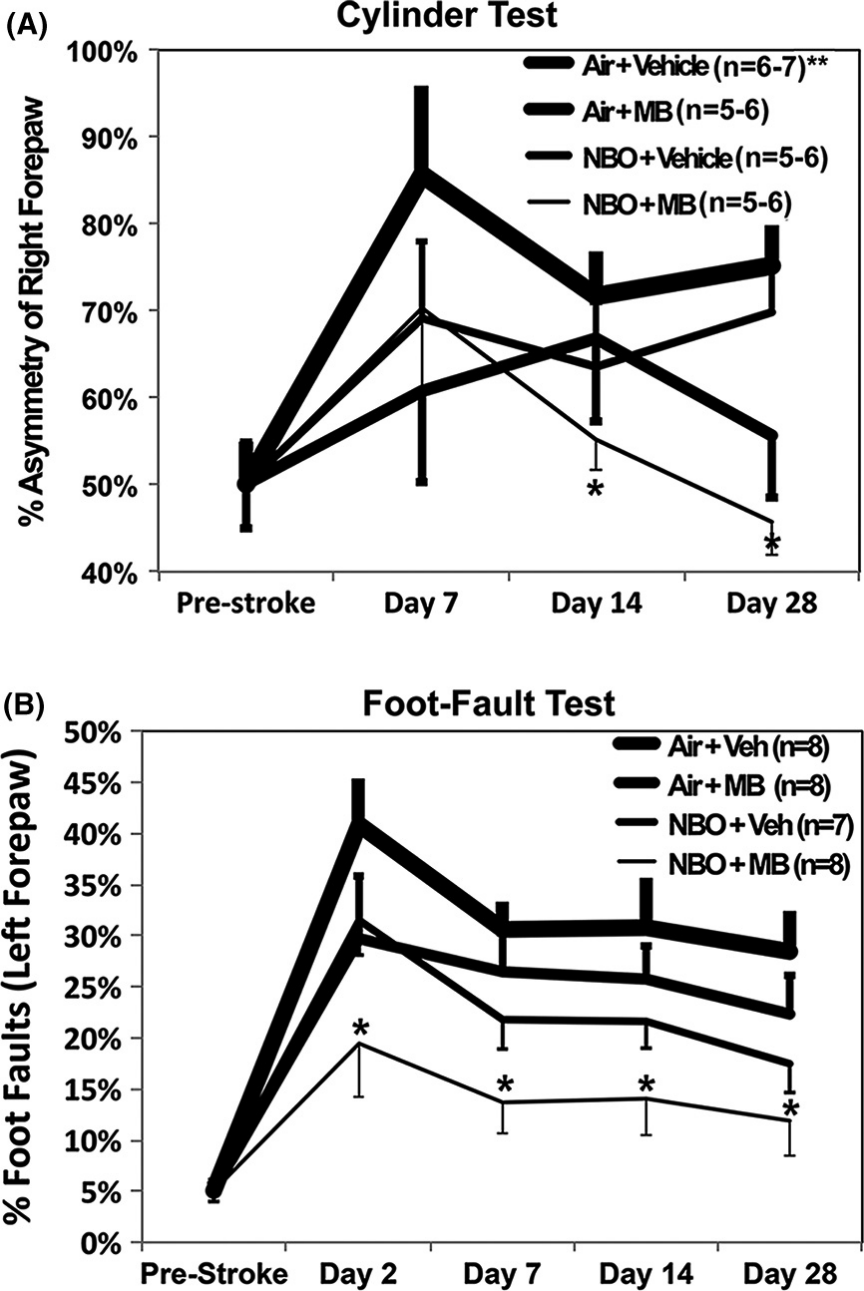
Functional assessment. (A) Percentage of right forepaw asymmetry in the four experimental groups (B) percentage of left forepaw foot‐faults (*P < 0.05). **The day 2 cylinder test values could not be scored due to animal inactivity.

Sensorimotor function using forelimb asymmetry and foot‐fault tests demonstrated a within‐group significant difference from baseline for all four groups (*P* = 0.03–0.001) measured at day 2 for the foot‐fault test, and day 7 for the cylinder test. On day 7, the percentage of right forepaw asymmetry for the Air + Vehicle, Air + MB, NBO + Vehicle, and NBO + MB groups were 86 ± 10, 61 ± 10, 69 ± 9, and 70 ± 10%, respectively. During the cylinder test (Fig. 3A), the Air + Vehicle control peaked in abnormal performance on day 7, and demonstrated mild functional recovery by days 14 and 28. By comparison, there was less increase in forepaw asymmetry for the Air + MB, NBO + Vehicle, and NBO + MB groups on day 7. On day 14, the percentage of right forepaw asymmetry for the Air + Vehicle, Air + MB, NBO + Vehicle, and NBO + MB groups were 72 ± 5, 67 ± 10, 63 ± 7, and 55 ± 4%, respectively. While the NBO + Vehicle and Air + MB groups performance approximated each other by day 14, the NBO + MB group demonstrated continuous improvement in performance that returned to normal values by days 14–28 across time (*P* = 0.005 vs. Air + Vehicle, repeated measures ANOVA). On day 28, the percentage of right forepaw asymmetry for the Air + Vehicle, Air + MB, NBO + Vehicle, and NBO + MB groups were 75 ± 5, 56 ± 7, 70 ± 7, and 46 ± 4%, respectively. A separate one‐way ANOVA of the means across groups for day 7, 14, and 28 of the cylinder test revealed a strong amongs‐group effect for day 28 (*P* = 0.004) resulting from the Air + MB versus Air + Vehicle (*P* = 0.035) and NBO + MB and Air + Vehicle (*P* = 0.006), and NBO + Vehicle versus NBO + MB (*P* = 0.05). Note that the NBO group showed a better asymmetry score than the MB group at 28 days, in contrast to other time points (please see Discussion).

The results of the sensorimotor assessment using the foot‐fault test (Fig. 3B) were similar to the cylinder test. On day 2, the percentage of left foot‐faults for the Air + Vehicle, Air + MB, NBO + Vehicle, and NBO + MB groups were 41 ± 6, 30 ± 4, 31 ± 3, and 20 ± 5%, respectively. The Air + Vehicle control peaked in abnormal performance on day 2, and showed gradual improvement over the following 28 days. On day 7, the percentage of left foot‐faults for the Air + Vehicle, Air + MB, NBO + Vehicle, and NBO + MB groups were 31 ± 4, 27 ± 3, 22 ± 3, and 14 ± 3%, respectively. The Air + MB and NBO + Vehicle groups were less abnormal than the control group on day 2, and demonstrated similar significant improvement over time compared to control (repeated measures ANOVA *P* = 0.046–0.08). By comparison, the NBO + MB group had the best outcome in performance on day 2, which was significantly different across time (day 2, 7, 14, and 28 from the Air + Vehicle group (*P* < 0.005) and NBO + MB vs. Air + MB (*P* = 0.016) and NBO + MB vs. NBO + Vehicle (*P* = 0.019)). On day 28, the percentage of left foot‐faults for the Air + Vehicle, Air + MB, NBO + Vehicle and NBO + MB groups were 28 ± 4, 22 ± 4, 17 ± 3, and 12 ± 3%, respectively.

## Discussion

This study applied longitudinal multimodal MRI and functional assessments to test the hypothesis that combined MB and NBO therapy further improves outcomes during the hyperacute to chronic phases of ischemic stroke compared to either treatment alone. The major findings were: (1) NBO + MB therapy showed a greater decrease in infarct volume compared to air + vehicle, NBO + vehicle group, but similar infarct volume compared to air + MB group, (2) Chronic NBO + MB therapy accelerated sensorimotor functional recovery compared to NBO or MB alone as demonstrated by the cylinder and foot‐fault tests, (3) Edema‐corrected infarct volumes on day 2 did not change significantly from final infarct volumes on day 28 for all four groups despite differences in functional recovery. These findings support the hypothesis that the combined NBO + MB improves functional outcome and reduces infarct volume compared to either treatment alone and these improvements extended up to 28 days.

### Methylene blue

Chronic low‐dose MB reduced lesion volume in the hyperacute to acute phase on day 2 that persisted up to 28 days in a 60‐min MCAO experimental stroke model. MB reduced infarct volume by 46% reduction compared to air group at 2 and 28 days. Our findings agree with and extend a previous study that found that single low‐dose MB (1 mg/kg given at 30–180 min after MCAO) prolonged the ischemic penumbra during occlusion in the hyperacute phase (Rodriguez et al. 2014), and another study that showed that single low‐dose MB (1 mg/kg given during reperfusion after 60 min MCAO) reduced infarct volume by 30% at 48 h compared to vehicle (Shen et al. 2013). NINDS stroke scores on days 2 and 7 also showed improved functional recovery (Shen et al. 2013). This study used more sophisticated functional assessments and further demonstrated that the improvement in infarct volumes and functional deficits persisted at least up to 28 days. We also showed that while chronic MB administration on days 2, 7, and 14 did not further decrease the final infarct volumes on day 28 compared to day 2, the intervention did maintain the reduced infarct volume, preventing further tissue damage, as well as improve functional recovery. To the best of our knowledge this is the first demonstration of positive efficacy of chronic MB treatment in experimental ischemic stroke. This positive neuroprotective effects are consistent with MB's energy‐enhancing and antioxidant properties and have been discussed previously (Shen et al. 2013; Rodriguez et al. 2014). By bypassing complexes I–III, the mitochondrial ATP generated by MB also has less superoxide free radical byproducts (Zhang et al. 2006; Bruchey and Gonzalez‐Lima 2008). MB could attenuate the negative effect of the hyperoxia by acting as an antioxidant to reduce free radical injury (Bruchey and Gonzalez‐Lima 2008), and by enhancing energy production at a time when there is a significant percentage of occluded capillaries (Clifton and Leikin 2003). Low‐dose MB suppresses inducible nitric oxide synthase, which plays a role in inflammatory pathways (Huang et al. 2015) and is overexpressed in transient brain ischemia and contributes to early necrosis (Iadecola et al. 1996).

### Normobaric hyperoxia

NBO treatment reduced lesion volume at 60 min after stroke compared to air group in the hyperacute phase (~60 min), but the improvement was not better than the air group at 2–28 days after stroke. NBO group showed only trends of improved behavioral function at 28 days compared to the air group. While there are many NBO treatment studies in experimental ischemic stroke, only a few used MRI to monitor tissue fate longitudinally before and during treatment. Singhal et al. studied permanent MCAO and found that NBO treatment minimized ADC abnormalities during the acute phase and reduced histological infarct volume at 48 h, as compared to the air group (Singhal et al. 2002a). Henninger et al. also studied the effects of NBO treatment in both permanent and transient MCAO models. NBO treatment reduced ADC abnormalities and stopped the progression of perfusion–diffusion mismatch during occlusion at the hyperacute phase, and significantly decreased histologically defined lesion volumes compared to the air group at 24 h (Henninger et al. 2007). Both these studies investigated lesion volumes with NBO treatment applied only during occlusion and used 1–2 day endpoints after stroke. These and other studies (Kim et al. 2005; Liu et al. 2006) only investigated NBO treatment during occlusion but not after reperfusion, presumably because NBO treatment after reperfusion could increase oxygen free radical damage. However, a few studies have found that NBO treatment in experimental ischemic stroke reduces free radical damage (Yuan et al. 2010) or does not increase oxygen‐related free radical stress markers (Flynn and Auer 2002; Singhal et al. 2002b; Yuan et al. 2014; Weaver and Liu 2015) even when given 1–7 h after reperfusion in combination with occlusion administration. It is possible that tissue perfusion does not fully return to normal after recannulation and thus a longer NBO course may improve outcomes. Indeed, there is also evidence that 10–20% of capillaries remain chronically occluded even after reperfusion (Liu et al. 2002).

We administered oxygen for about an hour each on day 2, 7, and 14. This protocol did not worsen nor improve lesion volume and function significantly likely because it was given during the nonhyperacute phase and its duration was brief. In sum, the efficacy of NBO treatment of ischemic stroke remains controversial and the outcomes likely depend on duration of treatment, stroke severity, stroke model and end points of the readouts. Further studies are needed before clinical translation of NBO treatment of ischemic stroke can be realized.

### Combination therapy

MB + NBO group showed a larger reduction in lesion volume in the hyperacute phase that sustained up to 28 days compared to the air and NBO groups. Although MB + NBO yielded similar infarct volumes as the MB group at 2 and 28 days, the MB + NBO group significantly outperformed the MB and other groups in functional recovery in foot‐fault scores, and trended toward significance in forelimb asymmetry scores by day 28. These findings suggest increased efficacy from the combined therapy *prior* to reperfusion, and during functional recovery. It is possible that NBO bought “time” and extended the treatment time window for MB to act. NBO and MB could act in synergy to further sustain ATP production in tissue at risk to support cell survival. Chronic MB + NBO administration could likely prevent further conversion of at risk tissue to infarct and thus help to sustain improvement over 28 days. The combination MB + NBO therapy may also have other pleiotropic effects via improved chronic oxygenation, inflammatory, apoptosis, autophagy and mitophagy‐related pathways that stimulate functional recovery.

Note that the NBO group showed a better asymmetry score than the MB group at 28 days, in contrast to other time points, which was unexpected. The pattern of the asymmetry score at 28 days also differed from that of the foot‐fault score. The cause of this minor discrepancy is unclear and requires further investigation. Nonetheless, the overall conclusion that MB + NBO group outperformed the other three groups in terms of behavioral scores remains valid.

### Differences between behavioral score and infarct volume

There is an apparent discrepancy between behavioral score and infarct volume as an outcome measure, namely that behavioral scores significantly improved with time, whereas infarct volumes did not significantly changes after day 2 for all treatment groups. Previous studies have reported differences in outcomes between behavioral score and infarct volume (Rogers et al. 1997; Dijkhuizen et al. 2003; Sicard et al. 2006). A possible explanation is that lesion volume had largely stopped evolving by about day 2, whereas function continues to improve. These improvements could be due to behavioral substitution, functional reorganization and/or functional compensation (Caleo 2015). For example, the rats may learn new strategies over time to compensate for the functional deficits. Recovery of function may also occur due to diaschisis (Feeney and Baron 1986) in which brain region outside of the injury that may be functionally depressed initially but recover over time and begin to function again. Nonetheless, both behavioral scores and infarct volumes showed MB + NBO generally yielded better outcomes compared to other treatment groups.

### Limitations and future perspectives

A limitation of this study is that MRI and behavior measurements were made only up to 28 days. It is possible that differences in measured parameters amongs groups could evolve further and it would be important to investigate functional reorganization in more chronic stage of stroke. This study focused on using MRI and behavioral scores to longitudinally evaluate treatment efficacies with the ultimate goal to translate to clinical trials. We did not investigate the molecular changes that contribute to the improved efficacy of the combined MB + NBO therapy because the MB and NBO mechanisms of action are already well known. We did not perform infarct volumes by histology because our previous studies have demonstrated that T2 lesion volumes are strongly correlated with histological infarct volume. This study also investigated only a single MCAO duration and its possible treatment efficacy could depend on stroke severity. Further work is also needed to better define the optimal dosing, frequency, and duration of combination therapy. Future studies should assess acute and chronic mitochondrial activity as well as immunohistochemistry markers that could justify faster functional recovery. These studies should use additional behavioral tests to confirm the measured motor and somatosensory recovery, and to assess other neurological functions (e.g., cognition). Overall, there is a lack of preclinical chronic stroke studies using MB and NBO, or combination therapy, with structural and functional neuroimaging, and behavioral testing. Other combination therapy with MB could also be explored. Low‐dose MB is also safe in humans, and clinical stroke trials using MB + NBO can thus be readily explored if it is further proven efficacious in animal stroke models. MB + NBO can potentially be administered by emergency responders onsite, enabling wider positive impact.

## Conclusions

Our results supported the hypothesis that combined MB + NBO treatment further reduces infarct volume and functional deficits compared to MB or NBO treatment alone, and the improvements persisted up to 28 days after stroke in rats.

## Conflict of Interest

The authors report no conflicts of interest.
